# First Record of a Suspected Human-Pathogenic *Borrelia* Species in Populations of the Bat Tick *Carios vespertilionis* in Sweden

**DOI:** 10.3390/microorganisms9051100

**Published:** 2021-05-20

**Authors:** Thomas G. T. Jaenson, Peter Wilhelmsson

**Affiliations:** 1 Evolutionary Biology Centre, Department of Organismal Biology, Uppsala University, 752 36 Uppsala, Sweden; thomas.jaenson@ebc.uu.se; 2 Department of Biomedical and Clinical Sciences, Division of Inflammation and Infection, Linköping University, 581 83 Linköping, Sweden; 3 Department of Clinical Microbiology, Region Jönköping County, 553 05 Jönköping, Sweden

**Keywords:** *Carios vespertilionis*, *Pipistrellus pygmaeus*, *Borrelia* sp. CPB1, relapsing fever, Sweden

## Abstract

The bat tick *Carios* *vespertilionis* has been reported from Sweden to occasionally feed on humans resulting in disease symptoms. The aim of this study was to investigate *C. vespertilionis* as a potential vector and reservoir of *Borrelia* species. In 2015 and 2018 in south-central Sweden, *C. vespertilionis* ticks were collected from a wooden bat box harboring Soprano pipistrelle bats, *Pipistrellus pygmaeus*. In addition, one *C. vespertilionis* tick found inside a house in southern Sweden in 2019 was collected. Ticks were screened for *Borrelia* spp. using a genus-specific quantitative PCR assay. The *Borrelia* species of the positive specimens were determined by conventional PCR followed by DNA sequencing and phylogenetic analyses. A total of 24% (22 of 92) of the analyzed *C. vespertilionis* ticks were *Borrelia*-positive. Phylogenetic analyses indicate that the bacteria belong to the relapsing fever group of borreliae; some of them appear to be identical with *Borrelia* sp. CPB1, a spirochete only found twice before—in the United Kingdom and in France. Our results also indicate a temporal and spatial distribution of this *Borrelia* species. Since *C. vespertilionis* occasionally bites humans, and since it exhibits a high prevalence of *Borrelia* bacteria, it is possible that it presents a risk of human disease. Further studies are needed to characterize *Borrelia* sp. CPB1 to determine if it is human-pathogenic and to determine if *C. vespertilionis* is a vector and/or reservoir of this agent.

## 1. Introduction

Many different species of putative or proven vertebrate-pathogenic parasites, bacteria, and viruses have been detected in bats and bat-associated ectoparasites, particularly ticks (Ixodida, Ixodidae, and Argasidae). Investigations of bat-associated microorganisms and viruses have drastically increased during the last two decades [1]. The main reasons for this are the severe outbreaks in humans by viruses related to or identical with bat-associated viruses, i.e., coronavirus disease 2019 (COVID-19), severe acute respiratory syndrome (SARS), Middle East respiratory syndrome (MERS), Ebola, bat lyssavirus rabies and Nipah [2,3,4,5,6,7].

There are more than 1400 species of bats [8], order Chiroptera, of which 19 species occur in Sweden [9]. Many bat species have synanthropic behavioral traits and benefit from using human habitations. For instance, nursery roosts of the Soprano pipistrelle bat, *Pipistrellus pygmaeus*, are in wall cavities and under roof coverings of houses, in tree holes, and in rock crevices [9,10,11]. *Pipistrellus pygmaeus* is distributed in western, central, eastern and southern Europe, including southern and south-central Sweden. According to Ahlén (2011), it is very common in southern Sweden northwards to River Dalälven and the coastal parts of the province of Gästrikland [11]. In the southern part of its Swedish range, this bat species occurs in all kinds of forests, gardens and parks, while to the north of Lake Mälaren it is mostly found in deciduous forests. Migration studies have shown that in recent years there have been many Soprano pipistrelle bats moving to wintering sites on the continent [11]. In Europe, populations of *P. pygmaeus* are often parasitized by the Round bat argas, also called the Short-legged bat tick, *Carios vespertilionis* (Ixodida, Argasidae) [12,13,14,15]. The resting places of these bats may be where humans are bitten by blood-feeding bat ectoparasites, such as the potentially pathogen-infected bat tick *C. vespertilionis.* This provides the opportunity for transmission of zoonotic pathogens [12,16,17].

*Carios vespertilionis* has a wide distribution range and is present in Africa, Asia and Europe [12,13,14,18], and it is usually host-specific to bats, particularly pipistrelles, but almost any bat species will serve as a host [13,19]. This tick species has been reported to be ‘highly aggressive’ [14] and may occasionally attack humans, dogs, and birds [12,16,17,18,19,20]. Hoogstraal (1956) writes: “Nymphs and adults on several occasions have attacked us in caves and we easily induce it to bite ourselves in the laboratory…On man mild itching resulting from a bite may persist for several weeks…It has been stated that this tick is a vector of a spirochaete of bats but reports of conclusive supporting evidence have thus far not been located” [19] (p. 106, p. 109). Furthermore, in the early summer of 1993 at Färentuna, province of Uppland, near Stockholm, Sweden, two persons experienced severe skin reactions with fever, ulceration, erythema, some swollen lymph glands, and oedema on their legs and arms after having been bitten by ticks in a bedroom. Shortly before that incident, unidentified bats had been roosting in the attic above the bedroom. One of us (TGTJ) morphologically identified the ticks as *C. vespertilionis* [16]. One of the two persons was eventually treated with penicillin, which cured the infection; the other person’s symptoms disappeared spontaneously without any pharmacological treatment. 

Only a few studies have investigated the presence of tick-associated microorganisms in *C. vespertilionis*. A total of 86% (29 of 34) of museum specimens of *C. vespertilionis* ticks collected between 1896 and 1994 in the United Kingdom contained DNA of *Borrelia burgdorferi* sensu lato as revealed by PCR [21]. *B. burgdorferi* s.l., the causative agent of the most common tick-borne human disease, Lyme borreliosis in the Holarctic Region, has also been detected in *C. vespertilionis* in southern Russia [22]. In France, *C. vespertilionis* ticks harbored a new genotype of the spotted fever group of rickettsiae called *Rickettsia* sp. AvBat, a new species of the *Ehrlichia canis* group—*Ehrlichia* sp. AvBat—and *Borrelia* sp. CPB1 [23], which is a member of the relapsing fever group of *Borrelia* and suspected to have caused a lethal *Borrelia* infection in a bat in the United Kingdom [24]. Other bacteria such as *Coxiella burnetii*, *Rickettsia* spp. and *Ehrlichia* spp., as well as the protozoan *Babesia vesperuginis*, and Issyk-Kul virus and Sokuluk virus, have been detected in *C. vespertilionis* ticks in Europe or Asia [21,25,26,27,28,29].

To our knowledge, the species of pathogens associated with bats and bat ticks in Sweden have not been previously investigated. The potential roles of *C. vespertilionis* as a reservoir and vector of bacteria, protozoan parasites and viruses are therefore worth investigating. A deeper knowledge about the biology of *C. vespertilionis* and its potential role as a reservoir and vector of harmful microorganisms is a prerequisite to better understand the biology of many bat species and to mitigate and potentially control certain zoonoses associated with this tick species. 

The aim of this study was to investigate the role of *C. vespertilionis* as a potential reservoir and vector of species in the *B. burgdorferi* s.l. complex and in the relapsing fever *Borrelia* complex.

## 2. Materials and Methods

### 2.1. Tick Collection

**Figure 1:**
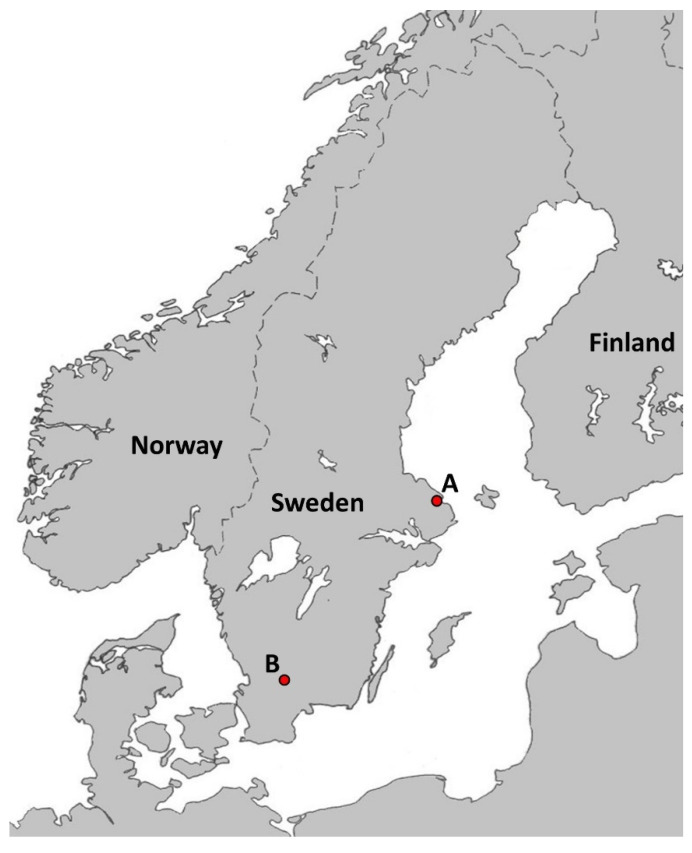
Map showing the localities where ticks were collected. (**A**) 91 ticks were collected from a water-filled tray placed below a wooden bat box located at Snesslinge (60°19.567 N, 18°15.067 E) in the province of Uppland, south-central Sweden. (**B**) One tick was collected inside a house located at Älmhult (56°32.720 N, 13°52.667 E) in the province of Småland, southern Sweden. The distance between A and B is 483 km.

In the summers of 2015 and 2018, at Snesslinge (60°19.567 N, 18°15.067 E, Figure 1), province of Uppland, south-central Sweden, 91 ticks were collected from a water-filled tray placed below a wooden bat box harboring an estimated number of 250–500 adult females and young, <2 months-old, Soprano pipistrelle bats, *P. pygmaeus*. All sampling occasions took place each morning from mid-June to mid-August in 2015 and in 2018. All ticks detected were put in numbered vials containing 80% ethanol. In addition, one tick specimen detected in July 2019 inside a house at Älmhult (56°32.720 N, 13°52.667 E, Figure 1), province of Småland, southern Sweden was also examined. The attic of this house was inhabited by bats of unknown species. The distance between Älmhult and Snesslinge is 483 km.

### 2.2. Tick Identification

All tick specimens were identified to developmental stage and species by morphological characteristics. A Leica Wild M10 stereomicroscope was used together with keys and illustrations in [13,19,30,31,32,33,34]. All ticks were also photographed dorsally and ventrally, and the length and width of each tick were measured, using a USB-microscope (Dino-Lite Long AM4013TL, AnMo Electronics Corp., Taiwan). Each tick was categorized into one of two categories, where + corresponds to “contains host blood” and—corresponds to “empty/no visible blood in the gut”.

Regarding the family Argasidae, we are aware that there is widespread disagreement concerning the genus-level classification and names of the genera in this family. Regarding the tick species, *Carios vespertilionis* (also known as *Argas vespertilionis*), found in this study, we followed the recent study of Mans et al. [35]. They showed that this species should be placed in the genus *Carios*, subfamily Ornithodorinae, family Argasidae. 

### 2.3. Nucleic Acid Extraction and cDNA Synthesis from Ticks

Collected ticks were homogenized individually by bead-beating in 2 mL safe-lock microcentrifuge tubes (Eppendorf AG, Hamburg, Germany) with a 5-mm stainless steel bead (Qiagen, Hilden, Germany) in 350 µL RLT buffer (Qiagen), supplemented with 1% 2-mercaptoethanol (Sigma-Aldrich, Stockholm, Sweden), using a TissueLyser II (Qiagen) for 2 min at 25 Hz. After centrifugation at 20,000× *g* for 3 min, 300 µL supernatant was transferred to new microcentrifuge tubes for total nucleic acid (NA) extraction, using MagAttract^®^ Viral RNA M48 kit (Qiagen) in a BioRobot M48 workstation (Qiagen), using a 65-µL elution volume. Each batch of 24 samples consisted of 22 ticks, one positive control (5 µL of *B. burgdorferi sensu stricto* B31 ATCC 35210 [10^8^ cells/mL]) and one negative control (H_2_O) that were extracted simultaneously.

The eluted NA was reverse-transcribed to cDNA using illustra™ Ready-to-Go RT-PCR Beads kit (GE Healthcare, Amersham Place, UK). Twenty microliters NA and 10 µL pd(N)6 random hexamer primers (0.25 µg/µL) were incubated for 5 min at 97 °C and then mixed with one RT-PCR bead dissolved in 20 µL RNase-free water. The mixture was incubated for 30 min at 42 °C, followed by 5 min at 97 °C, producing 50 µL cDNA.

### 2.4. Detection of Borrelia Bacteria and Determination of Species

Detection of *Borrelia* bacteria was done using a genus-specific TaqMan real-time PCR assay, as previously described [36]. The primers Borrelia-F and Borrelia-R and the probe Borrelia-P are designed to target the *Borrelia* spp. *16S* rRNA gene to amplify a 116-bp long amplicon (Table 1).

To determine *Borrelia* species of the samples positive in the TaqMan real-time PCR assay, a nested, conventional PCR assay using primers targeting the intergenic spacer region (IGS) between *5S* and *23S* rRNA genes (Table 1) was applied as previously described [37,38]. Samples that failed to produce PCR products with this assay were instead analyzed with primers targeting the IGS between *16S* and *23S* rRNA genes [39,40]. Tick samples, positive for *Borrelia* spp. in the TaqMan real-time PCR assay, which failed to produce PCR products with the 5S-23S IGS assay and the 16S-23S IGS assay, were denoted as ‘’untypeable’’. Samples positive for 16S-23S rRNA IGS were further analyzed with primers targeting the *16S* rRNA gene [41], and the *flaB* gene (this study, see below) (Table 1).

To further determine the *Borrelia* species of the samples positive with the 16S-23S IGS assay, another nested, conventional PCR assay using primers targeting the flagellin B gene (*flaB*), was developed. The primers flaB-F and flaB-R were designed to target the *Borrelia flaB* gene to amplify a 699-bp long amplicon of the species in the relapsing fever *Borrelia* complex (Table 1). A 50-µL reaction consisted of 10 µL 5× Phusion HF Buffer (Thermo Fisher Scientific, Waltham, MA, USA), 1 µL dNTP (10 mM), 2.5 µL of each primer (10 µM; Invitrogen; Table 1), 28.5 µL RNase-free water, 0.5 µL Phusion™ High-Fidelity DNA Polymerase (2 U/µL) (Thermo Fisher Scientific) and 5 µL cDNA template. The PCR reactions were performed on a MyCycler™ Thermal Cycler (Bio-Rad Laboratories, Inc., Hercules, CA, USA) using an activation step at 94 °C for 5 min, and 40 cycles of 94 °C for 1 min, 60 °C for 1 min, and 72 °C for 1 min, and finally one cycle of 72 °C for 10 min. An aliquot (5 µL) of the PCR product obtained in this assay was added to a second PCR mixture, which was prepared using the same volumes, concentrations, and amplification program as those for the first mixture, except with a different primer pair (flaB-Fn and flaB-Rn, Table 1).

### 2.5. Detection of Borrelia miyamotoi

Detection of *B. miyamotoi* was done using a species-specific TaqMan real-time PCR assay, as previously described [42]. The primers Bm_F and Bm_R, and the probe Bm_P, are designed to target the *B. miyamotoi* flagellin B gene (*flaB*) to amplify a 156-bp long amplicon [43] (Table 1). As a positive control, a synthetic plasmid containing the target sequence of the TaqMan real-time PCR assay was used. The plasmid contained the target sequence, spanning the nucleotides 510–665 of the *B. miyamotoi* flagellin (*flaB*) gene (GenBank: KT932823), synthesized and cloned into Eurofins standard vector carrying the ampicillin selection marker (Eurofins Genomics, Ebersberg, Germany).

### 2.6. Nucleotide Sequencing of PCR-Products and Phylogenetic Analysis 

Nucleotide sequencing of the PCR products amplified by conventional PCR assays to determine species of *Borrelia* was performed by Macrogen Inc. (Amsterdam, The Netherlands). All sequences were confirmed by sequencing both strands. The obtained chromatograms were initially edited and analyzed using BioEdit Software v7.0 (Tom Hall, Ibis Therapeutics, Carlsbad, CA, USA), and the sequences were examined using Basic Local Alignment Tool (BLAST). Phylogenetic trees were constructed with MEGA7 by neighbor-joining using Kimura 2-parameter and pairwise deletion with bootstrap value of 500 replicates. The scale bar of each phylogenetic tree corresponds to the number of substitutions per nucleotide site, and only values greater that 50% are shown in the trees. Sequences obtained have been deposited in GenBank with accession number MZ215741 for the 16S-23S IGS, MZ210080 for the 16S and MZ217187 for the flaB. One additional file shows all the aligned *Borrelia* sequences (see Document S1 in Supplementary Materials).

### 2.7. Statistical Analyses

Data were presented as percentages for categorical variables and as medians with interquartile range (IQR) for numerical variables. The categorical variables (e.g., developmental stage of the tick; season of tick collection, etc.) were analyzed using Yate’s corrected chi-square test, but when the expected frequency was <5 in at least one of the cells of the contingency table, Fisher’s exact test with a confidence interval (CI) of 95% was used instead. The numerical variables (i.e., the cycle threshold [Cq]-values obtained by the real-time PCR assay for the *Borrelia*-positive samples) were analyzed using Mann–Whitney test to compare Cq-values for the *Borrelia*-positive samples that could be determined to species with the *Borrelia*-positive samples that were denoted as ‘’untypeable’’. Statistical analyses were performed using GraphPad Prism version 8.0.0 for Windows (GraphPad Software, San Diego, CA, USA). *p*-Values ≤ 0.05 were considered statistically significant.

## 3. Results

### 3.1. Tick Collection and Tick Blood Engorgement

Between June and August in the summers of 2015 and 2018 in the province of Uppland, a total of 91 ticks (31 larvae, 48 nymphs and 12 adults) were collected from a water-filled tray placed below a wooden bat box. In 2015, 28 ticks (3 larvae, 19 nymphs and 6 adult ticks) were collected; n June: 5 nymphs and 3 adults; in July: 3 larvae, 6 nymphs and 3 adults; and in August: 8 nymphs. In 2018, 63 ticks (28 larvae, 29 nymphs and 6 adult ticks) were collected; in July: 10 larvae, 11 nymphs and 4 adults; and in August: 18 larvae, 18 nymphs and 2 adults. All ticks, except three nymphs, had visible blood in their guts. The tick collected in the province of Småland was an adult tick with visible blood in its gut. All ticks were microscopically identified as *C. vespertilionis*.

### 3.2. Prevalence of Borrelia Bacteria in the Ticks

Of all ticks collected from below the wooden bat box, 23.1% (21/91) were *Borrelia*-positive using the genus-specific real-time PCR assay. All *Borrelia*-positive ticks had visible blood in their guts. A significantly higher proportion of larvae (38.7%, 12/31) than nymphs (14.6%, 7/48) [χ^2^ = 4.75, *df* = 1, *P* = 0.029] was positive for *Borrelia* sp. There was no significant difference between the proportions of *Borrelia*-positive nymphs and *Borrelia*-positive adult ticks (16.7%, 2/12) and no significant difference between the proportion of *Borrelia*-positive larvae and that of *Borrelia*-positive adult ticks. No significant difference was detected between the proportion of *Borrelia*-positive ticks collected in 2015 (14.3%, 4/28) and that in 2018 (27.0%, 17/63). The adult tick collected in the house in Småland was *Borrelia*-positive based on the genus-specific real-time PCR assay. The prevalence of *Borrelia* bacteria in each tick developmental stage is shown in Table 2.

### 3.3. Phylogenetic Analysis of the Borrelia bacteria Detected in Ticks

Of all ticks tested, including the tick from Småland, 22 ticks were positive for genus-specific *Borrelia* DNA by real-time PCR analysis. Attempts to amplify the 5S-23S rRNA IGS from the positive samples were unsuccessful (Table 2).

**Figure 2:**
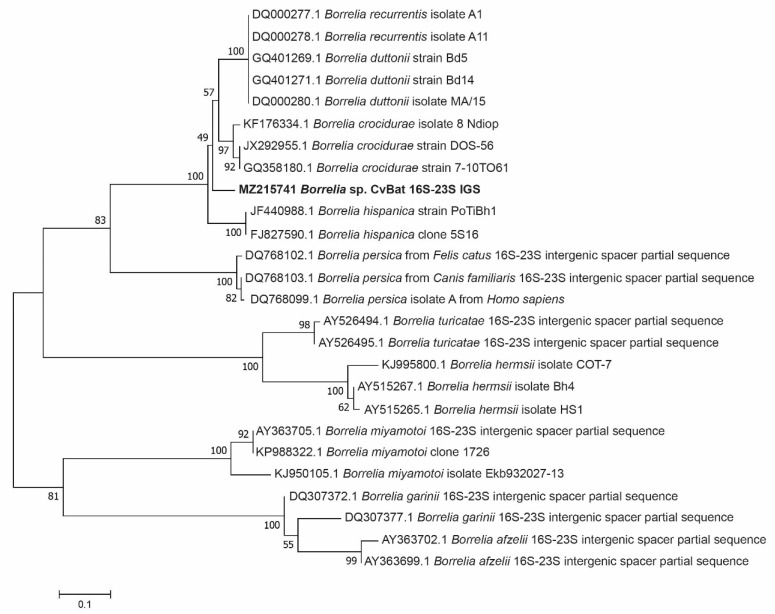
Phylogenetic tree based on 16S-23S intergenic spacer region sequences of *Borrelia* species. Sequences detected in our study (‘MZ215741 *Borrelia* sp. CvBat 16S-23S IGS’, *n* = 11) are highlighted in bold.

Amplification of the 16S-23S rRNA IGS, on the other hand, was successful in 11 samples, including the adult tick from Småland (Table 2). Subsequent sequencing of these amplicons showed that they were 100% identical to each other and will henceforth be collectively referred to as ‘’MZ215741 *Borrelia* sp. CvBat 16S-23S IGS’’ in Figure 2. The closest sequence to ‘’MZ215741 *Borrelia* sp. CvBat 16S-23S IGS’’, available in GenBank, was that of *Borrelia crocidurae* (accession no. CP003426), which showed 90.4% (482/533 bp) sequence identity.

**Figure 3:**
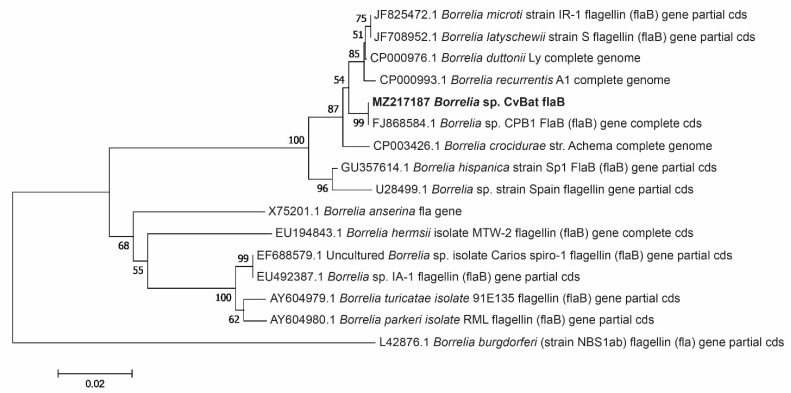
Phylogenetic tree based on *flaB* gene sequences of *Borrelia* species. Sequences detected in our study (‘‘MZ217187 *Borrelia* sp. CvBat flaB’’, *n* = 6) are highlighted in bold.

Amplification and sequencing of the *flaB* gene amplicon was successful in 6 samples, including the adult tick from Småland (Table 2). All sequences, referred to as ‘‘MZ217187 *Borrelia* sp. CvBat flaB’’ in Figure 3, were identical to each other and showed a 100% sequence identity (649/649) with the sequence of *Borrelia* sp. CPB1 (accession no. FJ868584).

**Figure 4:**
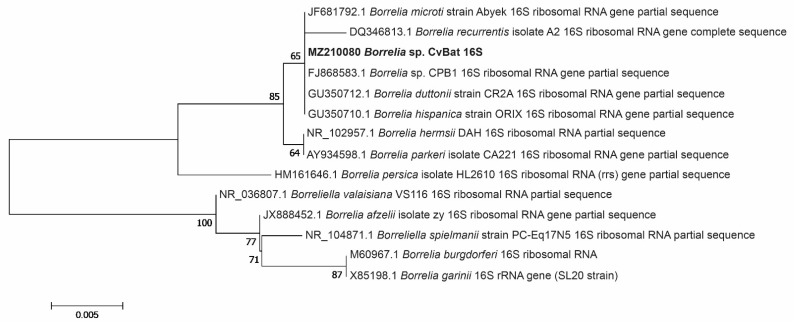
Phylogenetic tree based on *16S* rRNA gene sequences of *Borrelia* species. Sequences detected in our study (‘‘MZ210080 *Borrelia* sp. CvBat 16S’’, *n* = 4) are highlighted in bold.

Amplification and sequencing of the *16S* rRNA gene amplicon was successful in 4 samples (analysis of the adult tick from Småland was, however, not successful) (Table 2). All sequences, referred to as ‘‘MZ210080 *Borrelia* sp. CvBat 16S’’ in Figure 4, were identical to each other and showed 100% sequence identity (353/353) with those of *Borrelia* sp. CPB1 (accession no. FJ868583), *B. hispanica* (accession no. GU350710), *B. duttonii* (accession no. GU350712) and *B. microti* (accession no. JF681792).

Samples that resulted in a successful sequencing of the 16S-23S rRNA IGS had significantly lower Cq-values (*n* = 11, median 25.9, IQR 23.4–29.4) in the real-time PCR assay compared to samples that failed to produce PCR products with the 16S-23S IGS assay (*n* = 11, median 32.4, IQR 31.1–39.7, *p* = 0.003).

### 3.4. Prevalence of B. miyamotoi in the Ticks

All ticks, including the adult tick from Småland, were negative for *B. miyamotoi*.

## 4. Discussion

To our knowledge, this is the first study identifying *Borrelia* bacteria in ticks that have fed on blood from bats in Sweden. We recorded a high prevalence of *Borrelia* bacteria in populations of *C. vespertilionis* ticks. The phylogenetic analyses of ribosomal RNA genes and flagellin gene indicate that the bacteria belong to the relapsing fever group of borreliae; some of them appear to be identical to *Borrelia* sp. CPB1. *Borrelia* sp. CPB1 has been suspected to have caused a lethal *Borrelia* infection in a bat specimen in the United Kingdom [24]. The same *Borrelia* species was later identified in *C. vespertilionis* collected in a bat-infested house in France [23]. There is, to the best of our knowledge, no previous Swedish record of any *Borrelia* species from this tick species or from any bat species in Sweden. Our results also indicate a temporal and spatial distribution of this *Borrelia* species. It was detected in ticks collected at two locations separated by a distance of 480 km, and in ticks collected both in 2015 and 2018 and in 2019.

Except for one record of a specimen of *Otobius megnini*, the bat tick *C. vespertilionis* is the only argasid tick species present in Sweden and also the only bat-associated tick species recorded in Sweden [16]. *Carios vespertilionis* is widely distributed in Europe, southern Asia and North Africa, and all active stages are blood-feeding ectoparasites of bats of many different genera [34]. It inhabits locations where contact with humans may occur. Occasionally birds are hosts and sometimes even humans and dogs may be bitten [12,16,17,19,34]. The *Borrelia* spirochaetes are considered to have evolved as symbionts of ticks, particularly Argasidae, and are now endoparasites in mammals and birds, which act as amplifiers after having been bitten by infectious ticks [12].

All bat ticks investigated by us were negative for *B. miyamotoi.* However, *B. miyamotoi,* which is a member of the relapsing fever complex of *Borrelia* species, is present in Sweden [44,45]. *B. miyamotoi* was detected first in ixodid ticks in Japan [46]. Later it was revealed by Platonov et al. (2011) in Russia that this species is a human pathogen [47]. It is also present as a human pathogen in the United States and in Europe, including Sweden [48]. Contrary to the relapsing fever group of borreliae, which are vectored by argasid ticks, *B. miyamotoi* is transmitted by *Ixodes* ticks—in Sweden by *I. ricinus* and presumably by *I. persulcatus* [42,49]. Transovarial (vertical) transmission from female tick to her offspring and transstadial survival of the spirochaetes from larva to nymph to adult are common traits for *B. miyamotoi* in the *Ixodes* populations. It is possible that this new *Borrelia* sp. CPB1 is transovarially transmitted. There are at least two reasons for this: first, many other relapsing fever group borreliae are transovarially transmitted; second, the presence of a relatively high infection rate in all active stages—larvae, nymphs and adults—may indicate transstadial transmission. In contrast, the *B. burgdorferi* s.l. species are rarely if ever transovarially transmitted. Seven species in the *B. burgdorferi* s.l complex (*B. afzelii, B. burgdorferi sensu stricto, B. garinii, B. lusitaniae, B. spielmanii, B. turdi* and *B. valaisiana*) are present in the Swedish population of *I. ricinus* [37,45,50]. None of these species were detected in any of the *C. vespertilionis* ticks investigated in the present study. 

*Borrelia, Rickettsia, Ehrlichia, Bartonella, Coxiella* and *Babesia* species have previously been molecularly identified in *C. vespertilionis* from Europe [18,22,23,26,27,29]. It is often suggested that these putatively zoonotic microbes are vector-borne, i.e., tick-borne pathogens, presumably causing disease in bats and/or humans. However, this cannot be taken for granted. That a microorganism is tick-transmitted is doubtful if the tick specimen from which the DNA of the microbe was identified contained host blood. Nearly all of the ticks investigated by us contained visible host blood. Thus, our investigation neither shows that the *Borrelia* species found by us is tick-transmitted nor that it is pathogenic to bats or humans. While *B. miyamotoi* spirochaetes are transmitted to vertebrate hosts by the tick’s salivary fluid, other *Borrelia* relapsing fever spirochaetes are usually transmitted either in salivary fluid during blood ingestion by infectious larvae, nymphs and adult argasid ticks or in coxal fluid during and after feeding by nymphs and adult ticks. Since argasid larvae do not have any functioning coxal glands they can only transmit their relapsing fever spirochaetes via salivary fluid during blood feeding.

In the present study, a relatively large number of our *Borrelia*-positive specimens were not possible to identify by species; if they had been possible to identify, it is likely that some of them might have been *Borrelia* sp. CPB1. In 50% of the *Borrelia*-positive specimens as detected by the genus-specific quantitative PCR assay, attempts to amplify genes by conventional PCR assays were unsuccessful. These ticks contained, in general, a lower amount of *Borrelia* bacteria as indicated by a significantly higher Cq-value, compared to ticks containing a typeable *Borrelia* species. This may, at least partly, explain why PCR products, used to determine *Borrelia* species, were not amplified in the conventional PCR assays. There are two reasons why we used total nucleic acid (cDNA) and not DNA as template for PCR amplification: first, the use of cDNA in 16S rRNA based PCR-assays is more sensitive for detecting *Borrelia* than assays using DNA [45,51,52]; second, it allows us to investigate the presence of potential tick and/or bat associated RNA-viruses in the same material, which is planned to be done in subsequent work.

Deeper knowledge about the biology of *C. vespertilionis* and its role as a reservoir and vector of potentially harmful microorganisms is necessary to better understand the biology of many bat species and to mitigate and control potential zoonoses associated with this tick species. It is important to investigate the biology of the *Borrelia* species CPB1, its pathogenic potential to bats and humans, its geographic distribution, its host associations and the possibility of it being transmitted horizontally and/or vertically by *C. vespertilionis* and by other tick species such as *I. ricinus* and/or *I. vespertilionis*.

## 5. Conclusions

The examined tick populations had a high prevalence of species belonging to the relapsing fever *Borrelia* complex, but no species in the *B. burgdorferi* sensu lato complex were detected. The potential of *C. vespertilionis* as a reservoir and vector of *Borrelia* bacteria and other microorganisms needs to be further investigated.

## Figures and Tables

**Table 1:** Primers and probes used for molecular analysis of *Borrelia* bacteria.

Organism	Target	Oligo Name	Sequence (5’→3’)	Amplicon Length (bp)	Reference
*Borrelia* spp.	*16S* rRNA	Borrelia-F	GCTGAGTCACGAAAGCGTAG	116	[36]
		Borrelia-R	CACTTAACACGTTAGCTTCGGTA		
		Borrelia-P	FAM-CGCTGTAAACGATGCACACTTGGT-MGB		
	5S-23S rRNA IGS	B5S-23S_F	CTGCGAGTTCGCGGGAGA	225–266 ^a^	[38]
		B5S-23S_R	TCCTAGGCATTCACCATA		
		B5S-23S_Fn	GAGTTCGCGGGAGAGTAA		[37]
		B5S-23S_Rn	TAGGCATTCACCATAGACTCTT		
	16S-23S rRNA IGS	B16S-23S_F	GTATGTTTAGTGAGGGGGGTG	388–685 ^a^	[39,40]
		B16S-23S_R	GGATCATAGCTCAGGTGGTTAG		
		B16S-23S_Fn	AGGGGGGTGAAGTCGTAACAAG		
		B16S-23S_Rn	GTCTGATAAACCTGAGGTCGGA		
	*flaB*	flaB-F	CATCTGATGATGCTGCTGGT	699	This study
		flaB-R	TGTTTTGGAAAGCACCAAGA		
		flaB-Fn	GGGTGTTGCTGGGAAAATTA	672	
		flaB-Rn	TGGAAAGCACCAAGATTTGC		
	*16S* rRNA	M1	ACGATGCACACTTGGTGTTAA	357–358 ^a^	[41]
		M2	TCCGACTTATCACCGGCAGTC		
*B. miyamotoi*	*flaB*	Bm_F	AGAAGGTGCTCAAGCAG	156	[42]
		Bm_R	TCGATCTTTGAAAGTGACATAT		
		Bm_P	FAM-AGCACAACAGGAGGGAGTTCAAGC-BHQ1		

^a^ Amplicon length varies with the species. *Abbreviations*: FAM, 6-carboxy-fluorescine; MGB, minor groove binder; BHQ, black hole quencher; IGS, intergenic spacer.

**Table 2:** Number and prevalence of *Borrelia* bacteria detected in different stages of *Carios vespertilionis*.

Tick Developmental Stage	No. of Ticks Examined	No. (%) of *Borrelia*-Positive Ticks by Real-Time PCR	No. of Specimens with Successful Amplification of Gene Targets by Conventional PCR			
			5S-23S IGS	16S-23S IGS	*flaB*	*16S* rRNA
Larva	31	12 (38.7)	0	4	3	2
Nymph	48	7 (14.6)	0	4	1	1
Adult	13 ^a^	3 (23.1) ^a^	0	3 ^a^	2 ^a^	1
Total	92	22 (23.9)	0	11	6	4

^a^ One adult *C. vespertilionis* tick in this group was collected in the province of Småland.

## Data Availability

The data supporting the conclusions of this article are included within the article. Raw data can be shared with researchers upon a specific request. One additional file shows the aligned *Borrelia* sequences (Document S1).

## Supplementary Materials

The following are available online at https://www.mdpi.com/article/10.3390/microorganisms9051100/s1, Document S1: Aligned *Borrelia* nucleotide sequences based on PCR-products.
