# Supplementary material for: First Record of a Suspected Human-Pathogenic Borrelia Species in Populations of the Bat Tick Carios vespertilionis in Sweden
Source: Microorganisms. 2021 May 20;9(5):1100. doi: 10.3390/microorganisms9051100 (PMC8160990; doi:10.3390/microorganisms9051100)
Supplement: Supplementary file 1 [file microorganisms-09-01100-s001.zip › Document S1_changes.pdf]

Additional file 1. Aligned Borrelia nucleotide sequences based on PCR-products

The identifier line, which begins with '>', gives the name of the **sample ID_microorganism_gene target_tick species_developmental stage of tick_year of collection_sample location**

'Borrelia sp. CvBat 16S-23S IGS'

>176_Borrelia_16S-23S ribosomal RNA gene, partial sequence_Carios vespertilionis_nymph_2018_Uppland
AAGTGTGGCTGGATCACCTCCTTTCTAAGAGAAAGGTATTATTTAAAGGTAAATTATGTTTTAACTCTTCCTTTG
TCTTTTCAAAGACTGTTTTTGGGGGTTTAGCTCAGTTGGTTTAGAGCATCGGCTTTGCAAGCCGGGGGTCAAG
GGTTCGAGTCCCTTAACCTCCATTTATTTTAGGCCAAATAAATGGAGGTTATGATTAGAGGTAATGCTATGTTA
GTTGTAGCTAAATTTAAAATTGTTAAAAATACATTTTAATTTTATTATCTAAACAATGTGTTTTTATTGCGTCGTT
AATTAAGTAAAGTTGTTTACGTGTGGCAAGTCATGATAAGTGATCTTAATTTGATTTTGTTTTTTATTTTTTGTTT
GTATTCGTTATAATAAATTTTTGATTTTAAAGATGTGGGTTTAGGACTTGTAAATCACTAAGTGTAAGACATTTC
TTTAAATATTTGGATGTTTCTTTAAACG

>179_Borrelia_16S-23S ribosomal RNA gene, partial sequence_Carios vespertilionis_nymph_2018_Uppland
AAGTGTGGCTGGATCACCTCCTTTCTAAGAGAAAGGTATTATTTAAAGGTAAATTATGTTTTAACTCTTCCTTTG
TCTTTTCAAAGACTGTTTTTGGGGGTTTAGCTCAGTTGGTTTAGAGCATCGGCTTTGCAAGCCGGGGGTCAAG
GGTTCGAGTCCCTTAACCTCCATTTATTTTAGGCCAAATAAATGGAGGTTATGATTAGAGGTAATGCTATGTTA
GTTGTAGCTAAATTTAAAATTGTTAAAAATACATTTTAATTTTATTATCTAAACAATGTGTTTTTATTGCGTCGTT
AATTAAGTAAAGTTGTTTACGTGTGGCAAGTCATGATAAGTGATCTTAATTTGATTTTGTTTTTTATTTTTTGTTT
GTATTCGTTATAATAAATTTTTGATTTTAAAGATGTGGGTTTAGGACTTGTAAATCACTAAGTGTAAGACATTTC
TTTAAATATTTGGATGTTTCTT

>194_Borrelia_16S-23S ribosomal RNA gene, partial sequence_Carios vespertilionis_larva_2018_Uppland
AAGTGTGGCTGGATCACCTCCTTTCTAAGAGAAAGGTATTATTTAAAGGTAAATTATGTTTTAACTCTTCCTTTG
TCTTTTCAAAGACTGTTTTTGGGGGTTTAGCTCAGTTGGTTTAGAGCATCGGCTTTGCAAGCCGGGGGTCAAG
GGTTCGAGTCCCTTAACCTCCATTTATTTTAGGCCAAATAAATGGAGGTTATGATTAGAGGTAATGCTATGTTA
GTTGTAGCTAAATTTAAAATTGTTAAAAATACATTTTAATTTTATTATCTAAACAATGTGTTTTTATTGCGTCGTT
AATTAAGTAAAGTTGTTTACGTGTGGCAAGTCATGATAAGTGATCTTAATTTGATTTTGTTTTTTATTTTTTGTTT
GTATTCGTTATAATAAATTTTTGATTTTAAAGATGTGGGTTTAGGACTTGTAAATCACTAAGTGTAAGACATTTC
TTTAAATATTTGGATGTTTCTTTAAACGAG

>197_Borrelia_16S-23S ribosomal RNA gene, partial sequence_Carios vespertilionis_nymph_2018_Uppland
GTGTGGCTGGATCACCTCCTTTCTAAGAGAAAGGTATTATTTAAAGGTAAATTATGTTTTAACTCTTCCTTTGTC
TTTTCAAAGACTGTTTTTGGGGGTTTAGCTCAGTTGGTTTAGAGCATCGGCTTTGCAAGCCGGGGGTCAAGGG
TTCGAGTCCCTTAACCTCCATTTATTTTAGGCCAAATAAATGGAGGTTATGATTAGAGGTAATGCTATGTTAGTT
GTAGCTAAATTTAAAATTGTTAAAAATACATTTTAATTTTATTATCTAAACAATGTGTTTTTATTGCGTCGTTAAT
TAAGTAAAGTTGTTTACGTGTGGCAAGTCATGATAAGTGATCTTAATTTGATTTTGTTTTTTATTTTTTGTTTGTA
TTCGTTATAATAAATTTTTGATTTTAAAGATGTGGGTTTAGGACTTGTAAATCACTAAGTGTAAGACATTTCTTT
AAATATTTGGATGTTTCTTTAAACGAGTCCTAGTAAATTGGGACCAGGAGGAGTTGAA

>201_Borrelia_16S-23S ribosomal RNA gene, partial sequence_Carios vespertilionis_larva_2018_Uppland
AAGTGTGGCTGGATCACCTCCTTTCTAAGAGAAAGGTATTATTTAAAGGTAAATTATGTTTTAACTCTTCCTTTG
TCTTTTCAAAGACTGTTTTTGGGGGTTTAGCTCAGTTGGTTTAGAGCATCGGCTTTGCAAGCCGGGGGTCAAG
GGTTCGAGTCCCTTAACCTCCATTTATTTTAGGCCAAATAAATGGAGGTTATGATTAGAGGTAATGCTATGTTA
GTTGTAGCTAAATTTAAAATTGTTAAAAATACATTTTAATTTTATTATCTAAACAATGTGTTTTTATTGCGTCGTT
AATTAAGTAAAGTTGTTTACGTGTGGCAAGTCATGATAAGTGATCTTAATTTGATTTTGTTTTTTATTTTTTGTTT
GTATTCGTTATAATAAATTTTTGATTTTAAAGATGTGGGTTTAGGACTTGTAAATCACTAAGTGTAAGACATTTC
TTTAAATATTTGGATGTTTCTTTAAACGAGTCCTAGTAAATTGGGACCAGGAGGAG

>205_Borrelia_16S-23S ribosomal RNA gene, partial sequence_Carios vespertilionis_larva_2018_Uppland
GTGTGGCTGGATCACCTCCTTTCTAAGAGAAAGGTATTATTTAAAGGTAAATTATGTTTTAACTCTTCCTTTGTC
TTTTCAAAGACTGTTTTTGGGGGTTTAGCTCAGTTGGTTTAGAGCATCGGCTTTGCAAGCCGGGGGTCAAGGG
TTCGAGTCCCTTAACCTCCATTTATTTTAGGCCAAATAAATGGAGGTTATGATTAGAGGTAATGCTATGTTAGTT
GTAGCTAAATTTAAAATTGTTAAAAATACATTTTAATTTTATTATCTAAACAATGTGTTTTTATTGCGTCGTTAAT
TAAGTAAAGTTGTTTACGTGTGGCAAGTCATGATAAGTGATCTTAATTTGATTTTGTTTTTTATTTTTTGTTTGTA
TTCGTTATAATAAATTTTTGATTTTAAAGATGTGGGTTTAGGACTTGTAAATCACTAAGTGTAAGACATTTCTTT
AAATATTTGGATGTTTCTTTAAACGAGTCCTAGTAAATTGGGACCAGGAGGAGTTGAACCTCCGACC

>208_Borrelia_16S-23S ribosomal RNA gene, partial sequence_Carios vespertilionis_larva_2018_Uppland
AAGTGTGGCTGGATCACCTCCTTTCTAAGAGAAAGGTATTATTTAAAGGTAAATTATGTTTTAACTCTTCCTTTG
TCTTTTCAAAGACTGTTTTTGGGGGTTTAGCTCAGTTGGTTTAGAGCATCGGCTTTGCAAGCCGGGGGTCAAG
GGTTCGAGTCCCTTAACCTCCATTTATTTTAGGCCAAATAAATGGAGGTTATGATTAGAGGTAATGCTATGTTA
GTTGTAGCTAAATTTAAAATTGTTAAAAATACATTTTAATTTTATTATCTAAACAATGTGTTTTTATTGCGTCGTT
AATTAAGTAAAGTTGTTTACGTGTGGCAAGTCATGATAAGTGATCTTAATTTGATTTTGTTTTTTATTTTTTGTTT
GTATTCGTTATAATAAATTTTTGATTTTAAAGATGTGGGTTTAGGACTTGTAAATCACTAAGTGTAAGACATTTC
TTTAAATATTTGGATGTTTCTTTAAACGAGTCCTAGTAAATTGGGACCAGGAGGAGTTGAACCTCC

>218_Borrelia_16S-23S ribosomal RNA gene, partial sequence_Carios vespertilionis_adult_2015_Uppland
AAGTGTGGCTGGATCACCTCCTTTCTAAGAGAAAGGTATTATTTAAAGGTAAATTATGTTTTAACTCTTCCTTTG
TCTTTTCAAAGACTGTTTTTGGGGGTTTAGCTCAGTTGGTTTAGAGCATCGGCTTTGCAAGCCGGGGGTCAAG
GGTTCGAGTCCCTTAACCTCCATTTATTTTAGGCCAAATAAATGGAGGTTATGATTAGAGGTAATGCTATGTTA
GTTGTAGCTAAATTTAAAATTGTTAAAAATACATTTTAATTTTATTATCTAAACAATGTGTTTTTATTGCGTCGTT
AATTAAGTAAAGTTGTTTACGTGTGGCAAGTCATGATAAGTGATCTTAATTTGATTTTGTTTTTTATTTTTTGTTT
GTATTCGTTATAATAAATTTTTGATTTTAAAGATGTGGGTTTAGGACTTGTAAATCACTAAGTGTAAGACATTTC
TTTAAATATTTGGATGTTTCTTTAAACGAGTCCTAGTAAATTGGGACCAGGAGGAGTTGAACCTCC

>437_Borrelia_16S-23S ribosomal RNA gene, partial sequence_Carios vespertilionis_nymph_2015_Uppland
AGTGTGGCTGGATCACCTCCTTTCTAAGAGAAAGGTATTATTTAAAGGTAAATTATGTTTTAACTCTTCCTTTGT
CTTTTCAAAGACTGTTTTTGGGGGTTTAGCTCAGTTGGTTTAGAGCATCGGCTTTGCAAGCCGGGGGTCAAGG
GTTCGAGTCCCTTAACCTCCATTTATTTTAGGCCAAATAAATGGAGGTTATGATTAGAGGTAATGCTATGTTAG
TTGTAGCTAAATTTAAAATTGTTAAAAATACATTTTAATTTTATTATCTAAACAATGTGTTTTTATTGCGTCGTTA
ATTAAGTAAAGTTGTTTACGTGTGGCAAGTCATGATAAGTGATCTTAATTTGATTTTGTTTTTTATTTTTTGTTTG
TATTCGTTATAATAAATTTTTGATTTTAAAGATGTGGGTTTAGGACTTGTAAATCACTAAGTGTAAGACATTTCT
TTAAATATTTGGATGTTTCTTTAAACGAGTCCT

>181_Borrelia_16S-23S ribosomal RNA gene, partial sequence_Carios vespertilionis_adult_2015_Uppland
AGTGTGGCTGGATCACCTCCTTTCTAAGAGAAAGGTATTATTTAAAGGTAAATTATGTTTTAACTCTTCCTTTGT
CTTTTCAAAGACTGTTTTTGGGGGTTTAGCTCAGTTGGTTTAGAGCATCGGCTTTGCAAGCCGGGGGTCAAGG
GTTCGAGTCCCTTAACCTCCATTTATTTTAGGCCAAATAAATGGAGGTTATGATTAGAGGTAATGCTATGTTAG
TTGTAGCTAAATTTAAAATTGTTAAAAATACATTTTAATTTTATTATCTAAACAATGTGTTTTTATTGCGTCGTTA
ATTAAGTAAAGTTGTTTACGTGTGGCAAGTCATGATAAGTGATCTTAATTTGATTTTGTTTTTTATTTTTTGTTTG
TATTCGTTATAATAAATTTTTGATTTTAAAGATGTGGGTTTAGGACTTGTAAATCACTAAGTGTAAGACATTTCT
TTAAATATTTGGATGTTTCTTTAAACGAGTCCTAGTAAATTGGGACCAGGAGGAGTTGAACCTCC

>658_Borrelia_16S-23S ribosomal RNA gene, partial sequence_Carios vespertilionis_adult_2019_Småland
AAGTGTGGCTGGATCACCTCCTTTCTAAGAGAAAGGTATTATTTAAAGGTAAATTATGTTTTAACTCTTCCTTTG
TCTTTTCAAAGACTGTTTTTGGGGGTTTAGCTCAGTTGGTTTAGAGCATCGGCTTTGCAAGCCGGGGGTCAAG
GGTTCGAGTCCCTTAACCTCCATTTATTTTAGGCCAAATAAATGGAGGTTATGATTAGAGGTAATGCTATGTTA
GTTGTAGCTAAATTTAAAATTGTTAAAAATACATTTTAATTTTATTATCTAAACAATGTGTTTTTATTGCGTCGTT
AATTAAGTAAAGTTGTTTACGTGTGGCAAGTCATGATAAGTGATCTTAATTTGATTTTGTTTTTTATTTTTTGTTT
GTATTCGTTATAATAAATTTTTGATTTTAAAGATGTGGGTTTAGGACTTGTAAATCACTAAGTGTAAGACATTTC
TTTAAATATTTGGATGTTTCTTTAAACGAGTCCTAGTAAATTGGGACCAGGAGGAGTTGAACCTCC

'Borrelia sp. CvBat 16S'

>197_Borrelia_16S ribosomal RNA gene, partial sequence_Carios vespertilionis_nymph_2018_Uppland
ACGATGCACACTTGGTGTTAATCGAGAGGTTAGTACCGAAGCTAACGTGTTAAGTGTGCCGCCTGGGGAGTAT
GCTCGCAAGAGTGAAACTCAAAGGAATTGACGGGGGCCCGCACAAGCGGTGGAGCATGTGGTTTAATTCGAT
GATACGCGAGGAACCTTACCAGGGCTTGACATATACAGGATGTAGTTAGAGATAACTATTCCCCGTTTGGGGT
CTGTATACAGGTGCTGCATGGTTGTCGTCAGCTCGTGCTGTGAGGTGTTGGGTTAAGTCCCGCAACGAGCGCA
ACCCTTATTGTCTGTTACCAGCATGTAAAGATGGGGACTCAGACGAGACTGCCGGTGATAAG

>201_Borrelia_16S ribosomal RNA gene, partial sequence_Carios vespertilionis_larva_2018_Uppland
ACGATGCACACTTGGTGTTAATCGAGAGGTTAGTACCGAAGCTAACGTGTTAAGTGTGCCGCCTGGGGAGTAT
GCTCGCAAGAGTGAAACTCAAAGGAATTGACGGGGGCCCGCACAAGCGGTGGAGCATGTGGTTTAATTCGAT
GATACGCGAGGAACCTTACCAGGGCTTGACATATACAGGATGTAGTTAGAGATAACTATTCCCCGTTTGGGGT
CTGTATACAGGTGCTGCATGGTTGTCGTCAGCTCGTGCTGTGAGGTGTTGGGTTAAGTCCCGCAACGAGCGCA
ACCCTTATTGTCTGTTACCAGCATGTAAAGATGGGGACTCAGACGAGACTGCCGGTGATAAG

>208_Borrelia_16S ribosomal RNA gene, partial sequence_Carios vespertilionis_larva_2018_Uppland
ACGATGCACACTTGGTGTTAATCGAGAGGTTAGTACCGAAGCTAACGTGTTAAGTGTGCCGCCTGGGGAGTAT
GCTCGCAAGAGTGAAACTCAAAGGAATTGACGGGGGCCCGCACAAGCGGTGGAGCATGTGGTTTAATTCGAT
GATACGCGAGGAACCTTACCAGGGCTTGACATATACAGGATGTAGTTAGAGATAACTATTCCCCGTTTGGGGT
CTGTATACAGGTGCTGCATGGTTGTCGTCAGCTCGTGCTGTGAGGTGTTGGGTTAAGTCCCGCAACGAGCGCA
ACCCTTATTGTCTGTTACCAGCATGTAAAGATGGGGACTCAGACGAGACTGCCGGTGATAAG

>218_Borrelia_16S ribosomal RNA gene, partial sequence_Carios vespertilionis_adult_2015_Uppland
ACGATGCACACTTGGTGTTAATCGAGAGGTTAGTACCGAAGCTAACGTGTTAAGTGTGCCGCCTGGGGAGTAT
GCTCGCAAGAGTGAAACTCAAAGGAATTGACGGGGGCCCGCACAAGCGGTGGAGCATGTGGTTTAATTCGAT
GATACGCGAGGAACCTTACCAGGGCTTGACATATACAGGATGTAGTTAGAGATAACTATTCCCCGTTTGGGGT
CTGTATACAGGTGCTGCATGGTTGTCGTCAGCTCGTGCTGTGAGGTGTTGGGTTAAGTCCCGCAACGAGCGCA
ACCCTTATTGTCTGTTACCAGCATGTAAAGATGGGGACTCAGACGAGACTGCCGGTGATAAG

'Borrelia sp. CvBat flaB'

>197_Borrelia_flaB gene, partial sequence_Carios vespertilionis_nymph_2018_Uppland
GGAAAATTAACGCTCAAATTAGAGGGTTATCCCAAGCTTCAAGAAATACTTCAAAGGCAATAAATTTTATTCAA
ACAACAGAAGGAAATTTGGATGAAGTAGAGAAAGTGTTGGTGAGAATGAAAGAGCTTGCTGTTCAATCTGGT
AATGGTACATATTCAGATGCAGACAGAGGTTCTATTCAGATTGAAGTTGAGCAACTTACAGATGAGATCAATA
GAATTGCTGATCAGGCACAATATAACCATATGCATATGTTATCTAATAGATCATCTGCTGAGCATGTAAGAACA
GCTGAAGAGCTTGGAATGCAACCTGTAAAGATTAATACACCAGCATCATTATCTGGCTCACAAGCTTCATGGAC
ATTAAGAGTACATGTTGGTGCAAATCAAGATGAAGCAATTGCTGTTAATATTTATGCAGCTAATGTTGCAAATC
TTTTTTCGGGTGAGGGTGCTCAACAAGTAGCTCCAGCTCAAGAGGGTGCACAGCAAGAAGGAGCACAAGCAG
CTCCAGCTCCAGCAGCAGCTCCAGCTCAAGGTGGTGTTAACTCTCCAGTTAATGTTACAACTGCTGTTGATGCT
AATATGTCACTTACAAAGATAGAAGATGCTATTAGAATGGTAACTGATCAAAGAGCAAATCT

>201_Borrelia_flaB gene, partial sequence_Carios vespertilionis_larva_2018_Uppland
AAATTAACGCTCAAATTAGAGGGTTATCCCAAGCTTCAAGAAATACTTCAAAGGCAATAAATTTTATTCAAACA
ACAGAAGGAAATTTGGATGAAGTAGAGAAAGTGTTGGTGAGAATGAAAGAGCTTGCTGTTCAATCTGGTAAT
GGTACATATTCAGATGCAGACAGAGGTTCTATTCAGATTGAAGTTGAGCAACTTACAGATGAGATCAATAGAA
TTGCTGATCAGGCACAATATAACCATATGCATATGTTATCTAATAGATCATCTGCTGAGCATGTAAGAACAGCT
GAAGAGCTTGGAATGCAACCTGTAAAGATTAATACACCAGCATCATTATCTGGCTCACAAGCTTCATGGACATT
AAGAGTACATGTTGGTGCAAATCAAGATGAAGCAATTGCTGTTAATATTTATGCAGCTAATGTTGCAAATCTTT
TTTCGGGTGAGGGTGCTCAACAAGTAGCTCCAGCTCAAGAGGGTGCACAGCAAGAAGGAGCACAAGCAGCTC
CAGCTCCAGCAGCAGCTCCAGCTCAAGGTGGTGTTAACTCTCCAGTTAATGTTACAACTGCTGTTGATGCTAAT
ATGTCACTTACAAAGATAGAAGATGCTATTAGAATGGTAACTGATCAAAGAGCAAATCTTG

>205_Borrelia_flaB gene, partial sequence_Carios vespertilionis_larva_2018_Uppland
GGAAAATTAACGCTCAAATTAGAGGGTTATCCCAAGCTTCAAGAAATACTTCAAAGGCAATAAATTTTATTCAA
ACAACAGAAGGAAATTTGGATGAAGTAGAGAAAGTGTTGGTGAGAATGAAAGAGCTTGCTGTTCAATCTGGT
AATGGTACATATTCAGATGCAGACAGAGGTTCTATTCAGATTGAAGTTGAGCAACTTACAGATGAGATCAATA
GAATTGCTGATCAGGCACAATATAACCATATGCATATGTTATCTAATAGATCATCTGCTGAGCATGTAAGAACA
GCTGAAGAGCTTGGAATGCAACCTGTAAAGATTAATACACCAGCATCATTATCTGGCTCACAAGCTTCATGGAC
ATTAAGAGTACATGTTGGTGCAAATCAAGATGAAGCAATTGCTGTTAATATTTATGCAGCTAATGTTGCAAATC
TTTTTTCGGGTGAGGGTGCTCAACAAGTAGCTCCAGCTCAAGAGGGTGCACAGCAAGAAGGAGCACAAGCAG
CTCCAGCTCCAGCAGCAGCTCCAGCTCAAGGTGGTGTTAACTCTCCAGTTAATGTTACAACTGCTGTTGATGCT
AATATGTCACTTACAAAGATAGAAGATGCTATTAGAATGGTAACTGATCAAAGAGCAAAT

>208_Borrelia_flaB gene, partial sequence_Carios vespertilionis_larva_2018_Uppland

GGAAAATTAACGCTCAAATTAGAGGGTTATCCCAAGCTTCAAGAAATACTTCAAAGGCAATAAATTTTATTCAA
ACAACAGAAGGAAATTTGGATGAAGTAGAGAAAGTGTTGGTGAGAATGAAAGAGCTTGCTGTTCAATCTGGT
AATGGTACATATTCAGATGCAGACAGAGGTTCTATTCAGATTGAAGTTGAGCAACTTACAGATGAGATCAATA
GAATTGCTGATCAGGCACAATATAACCATATGCATATGTTATCTAATAGATCATCTGCTGAGCATGTAAGAACA
GCTGAAGAGCTTGGAATGCAACCTGTAAAGATTAATACACCAGCATCATTATCTGGCTCACAAGCTTCATGGAC
ATTAAGAGTACATGTTGGTGCAAATCAAGATGAAGCAATTGCTGTTAATATTTATGCAGCTAATGTTGCAAATC
TTTTTTCGGGTGAGGGTGCTCAACAAGTAGCTCCAGCTCAAGAGGGTGCACAGCAAGAAGGAGCACAAGCAG
CTCCAGCTCCAGCAGCAGCTCCAGCTCAAGGTGGTGTTAACTCTCCAGTTAATGTTACAACTGCTGTTGATGCT
AATATGTCACTTACAAAGATAGAAGATGCTATTAGAATGGTAACTGATCAAAGAGCAAATCT

>218_Borrelia_flaB gene, partial sequence_Carios vespertilionis_adult_2015_Uppland
AAAATTAACGCTCAAATTAGAGGGTTATCCCAAGCTTCAAGAAATACTTCAAAGGCAATAAATTTTATTCAAAC
AACAGAAGGAAATTTGGATGAAGTAGAGAAAGTGTTGGTGAGAATGAAAGAGCTTGCTGTTCAATCTGGTAA
TGGTACATATTCAGATGCAGACAGAGGTTCTATTCAGATTGAAGTTGAGCAACTTACAGATGAGATCAATAGA
ATTGCTGATCAGGCACAATATAACCATATGCATATGTTATCTAATAGATCATCTGCTGAGCATGTAAGAACAGC
TGAAGAGCTTGGAATGCAACCTGTAAAGATTAATACACCAGCATCATTATCTGGCTCACAAGCTTCATGGACAT
TAAGAGTACATGTTGGTGCAAATCAAGATGAAGCAATTGCTGTTAATATTTATGCAGCTAATGTTGCAAATCTT
TTTTCGGGTGAGGGTGCTCAACAAGTAGCTCCAGCTCAAGAGGGTGCACAGCAAGAAGGAGCACAAGCAGCT
CCAGCTCCAGCAGCAGCTCCAGCTCAAGGTGGTGTTAACTCTCCAGTTAATGTTACAACTGCTGTTGATGCTAA
TATGTCACTTACAAAGATAGAAGATGCTATTAGAATGGTAACTGATCAAAGAGCAAATCT

>658_Borrelia_flaB gene, partial sequence_Carios vespertilionis_adult_2019_Småland
GGAAAATTAACGCTCAAATTAGAGGGTTATCCCAAGCTTCAAGAAATACTTCAAAGGCAATAAATTTTATTCAA
ACAACAGAAGGAAATTTGGATGAAGTAGAGAAAGTGTTGGTGAGAATGAAAGAGCTTGCTGTTCAATCTGGT
AATGGTACATATTCAGATGCAGACAGAGGTTCTATTCAGATTGAAGTTGAGCAACTTACAGATGAGATCAATA
GAATTGCTGATCAGGCACAATATAACCATATGCATATGTTATCTAATAGATCATCTGCTGAGCATGTAAGAACA
GCTGAAGAGCTTGGAATGCAACCTGTAAAGATTAATACACCAGCATCATTATCTGGCTCACAAGCTTCATGGAC
ATTAAGAGTACATGTTGGTGCAAATCAAGATGAAGCAATTGCTGTTAATATTTATGCAGCTAATGTTGCAAATC
TTTTTTCGGGTGAGGGTGCTCAACAAGTAGCTCCAGCTCAAGAGGGTGCACAGCAAGAAGGAGCACAAGCAG
CTCCAGCTCCAGCAGCAGCTCCAGCTCAAGGTGGTGTTAACTCTCCAGTTAATGTTACAACTGCTGTTGATGCT
AATATGTCACTTACAAAGATAGAAGATGCTATTAGAATGGTAACTGATCAAAGAGCAAATCT
